# Veno-Arterial Extracorporeal Membrane Oxygenation for Patients Undergoing Acute Type A Aortic Dissection Surgery: A Six-Year Experience

**DOI:** 10.3389/fcvm.2021.652527

**Published:** 2021-05-17

**Authors:** Jun-yi Hou, Chun-sheng Wang, Hao Lai, Yong-xin Sun, Xin Li, Ji-li Zheng, Huan Wang, Jing-chao Luo, Guo-wei Tu, Zhe Luo

**Affiliations:** ^1^Department of Critical Care Medicine, Zhongshan Hospital, Fudan University, Shanghai, China; ^2^Department of Cardiovascular Surgery, Zhongshan Hospital, Fudan University, Shanghai, China; ^3^Department of Nursing, Zhongshan Hospital, Fudan University, Shanghai, China; ^4^Department of Critical Care Medicine, Xiamen Branch, Zhongshan Hospital, Fudan University, Xiamen, China

**Keywords:** acute type A aortic dissection, veno-arterial extracorporeal membrane oxygenation, cardiogenic shock, aortic surgery, acute

## Abstract

**Objectives:** Acute type A aortic dissection (aTAAD) is usually lethal without emergency surgery. Although veno-arterial extracorporeal membrane oxygenation (VA-ECMO) is widely used in patients with cardiogenic shock following cardiac surgery, VA-ECMO support following aTAAD surgery has not been well-described. Based on our 6-year experience, we aimed to retrospectively analyze risk factors, application and timing of VA-ECMO, and outcomes in aTAAD patients.

**Methods:** In this retrospective, single-center study, we enrolled adult patients who underwent aTAAD surgery from January 2014 to December 2019 and were supported with VA-ECMO. Patients were divided into two groups according to whether or not they were successfully weaned from VA-ECMO. Preoperative, intraoperative and postoperative variables were assessed and analyzed. Outcomes of the patients were followed up until discharge.

**Results:** Twenty-seven patients who received aTAAD surgery with VA-ECMO support were included in the study. Nine patients (33.3%) were successfully weaned from VA-ECMO. The median VA-ECMO support time and length of hospital stay in the successfully weaned group were significantly longer than in the group could not be successfully weaned (192 [111–327] vs. 55 [23–95] h, *p* < 0.01; 29 [18–40] vs. 4 [3–8] days, *p* < 0.01). Overall in-hospital mortality was 81.5%. The main causes of death were bleeding (37%), neurological complications (15%), and multiple organ dysfunction syndrome (15%). Preoperative levels of creatine kinase-MB (CK-MB) were lower in patients who were successfully weaned from VA-ECMO than in the failed group (14 [6–30] vs. 55 [28–138] U/L, *p* < 0.01). Postoperative peak levels of CK-MB, cardiac troponin T, lactate dehydrogenase, and lactate were significantly lower in the successful group than in the failed group.

**Conclusion:** Postoperative VA-ECMO support was rarely used in aTAAD patients. Our study showed that VA-ECMO can be considered as a salvage treatment in aTAAD patients, despite the high rate of complications and mortality.

## Introduction

Acute type A aortic dissection (aTAAD) is a cardiovascular emergency associated with the formation of a false lumen in the media, caused by intimal weakness or tear. Blood surges through the false lumen and enlarges the tear at the proximal, distal, or both ends (1). Because of its high mortality rate, aTAAD is one of the most urgent surgical emergencies in cardiac surgical patients. Despite significant improvements in surgical techniques, cardiopulmonary bypass (CPB) practices, cerebral protection procedures, and perioperative management data published in the International Registry of Acute Aortic Dissection (IRAD) show that the mortality rate in aTAAD surgery is still ~20% (2).

The heart is one of the organs most commonly affected by aTAAD, which can lead to cardiac tamponade, acute severe aortic regurgitation, and/or coronary artery involvement. Preoperative coronary artery dissection is usually associated with acute myocardial infarction and heart failure. A previous study showed that mortality associated with aTAAD involving the coronary artery was as high as 20% (3). Poor myocardial protection during surgery can also lead to myocardial ischemia or ischemia-reperfusion injury. Perioperative multiorgan malperfusion can also lead to uncorrectable acidosis and end organ dysfunction, which contribute to the extremely high mortality (4, 5).

Veno-arterial extracorporeal membrane oxygenation (VA-ECMO), which provides temporary circulatory support for critically ill patients with refractory cardiogenic shock and cardiac arrest, can be used as a bridge to myocardial recovery in aTAAD patients (6, 7). Since ventricular assist devices are not yet available in China, our cardiovascular center has routinely used VA-ECMO to treat patients with CS. The use of VA-ECMO in aTAAD patients with cardiogenic shock has not, however, been well-documented although the prognosis of patients weaned from VA-ECMO support after aTAAD surgery is known to be poor. In the present study, we aimed to investigate the use and timing of VA-ECMO, risk factors and outcomes in aTAAD patients.

## Materials and Methods

### Patient and Study Design

In this retrospective, single-center study, we reviewed the records of adult patients who received VA-ECMO support after aTAAD surgery at Zhongshan Hospital, Fudan University (Shanghai, China) from January 2014 to December 2019. Exclusion criteria included: an age <18 years old or pregnancy. Zhongshan Hospital performs over 150 aTAAD surgeries per year, including ascending aortic and hemi- or total-arch replacement, with or without concomitant surgical treatment of the aortic root, as well as elephant trunk stent procedures for the descending aorta. The study was approved by the Ethics Committee of Zhongshan Hospital.

Patients were divided into two groups according to whether the VA-ECMO was successfully removed or not (patients were defined as successfully weaned from VA-ECMO if they survived for longer than 48 h after VA-ECMO explantation) (8, 9). Baseline variables, which included age, gender, body mass index, comorbidities, laboratory tests, and operative characteristics, together with outcome variables, which included VA-ECMO support time, VA-ECMO weaning rate, mechanical ventilation time, length of stay in intensive care unit (ICU), length of hospital stay, complications, and in-hospital mortality, were compared between the two groups. All data were collected from the patients' hospital records by two residents (H-W and JY-H).

### Surgical Procedures

All aTAAD patients underwent emergency surgery, unless the patient refused surgery or had preoperative neurological complications. Surgical repair was performed under CPB and deep hypothermic circulatory arrest. In some patients, replacement of the ascending and proximal arch was sufficient, but when the intimal tear was in the aortic arch, total arch replacement was performed. We routinely used unilateral selective antegrade cerebral perfusion with deep hypothermic circulatory arrest as a cerebral protection strategy during emergent surgical repair of aTAAD. We used continuous cerebral near-infrared spectroscopy to monitor brain oxygenation during surgery.

### Timing of VA-ECMO Implantation

The decision to use VA-ECMO was made by the cardiac surgeon in the operating room or by the intensivist in the cardiac surgery ICU. Indications for VA-ECMO therapy included difficulty weaning from CPB or postoperative refractory cardiogenic shock despite adequate volumes and high doses of inotropes such as norepinephrine, dobutamine, epinephrine, and milrinone. A femoral venous cannula placed from the femoral vein to the right atrium was used as the VA-ECMO venous cannula. The femoral artery is most commonly used for arterial catheterization in adult patients, but this puts the aTAAD patients at risk of developing Harlequin syndrome. Because of this, we routinely used right axillary artery catheterization in aTAAD patients with refractory hypoxemia.

### Management During VA-ECMO Support

In the initial stage of VA-ECMO, the target mean arterial pressure was maintained at ≥60 mmHg, thus, ensuring tissue perfusion without excessive increase of afterload. Serum lactate level was used as an indicator of tissue hypoperfusion and has been proved to predict outcomes of cardiogenic shock associated with organ failure (10). Cardiac structure and function, and hemodynamic conditions were routinely assessed by transesophageal and/or transthoracic echocardiography.

As soon as the VA-ECMO guidewire was implanted, heparin (1 mg/kg) was given intravenously. During VA-ECMO support, heparin was used as an anticoagulant. Active clotting time was maintained at 180–200 s or activated partial thromboplastin time was maintained at 50–80 s in patients with low bleeding risk. In patients with high bleeding risk, active clotting time was maintained at 160 s. Platelets were transfused when the patient's platelet count fell below 50 × 10^9^/L (11).

A protective lung ventilation strategy was used, including an initial tidal volume of 6 mL/kg of ideal body weight, a positive end expiratory pressure of 5–10 cm H_2_O, a respiratory rate of 10–12 times/min and fraction of inspired oxygen no more than 50%. All patients received remifentanil and midazolam to achieve target sedation and underwent daily awakening trials. We routinely monitored cerebral oxygen saturation and carried out a physical examination of the nervous system (12, 13).

When the primary disease was well-treated, the hemodynamics was stable and the tissue perfusion was satisfactory, the flow rate was gradually reduced to 1.5 L/min. Removal of ECMO was considered if echocardiography indicated that the left ventricular outflow tract velocity time integral was >10 cm, the lateral mitral annulus peak systolic velocity was >6 cm/s and the left ventricular ejection fraction was >25%(14).

### Statistical Analysis

Continuous data are expressed as mean ± standard deviation (SD), if normally distributed, or median (IQR), if not normally distributed. For continuous variables, the normality of distribution was evaluated using the Kolmogorov–Smirnov test. Categorical variables are summarized as percentages (%). Categorical variables were analyzed using the χ^2^ test or Fisher's exact methods and quantitative variables were analyzed using the Student's *t-*test or Mann–Whitney *U*-test as appropriate. No adjustment was made for multiplicity. Log-rank testing and Kaplan–Meier survival curves were used to analyze survival. Statistical significance was defined as *p* < 0.05. Statistical analysis was performed using SPSS software (version 19.0; SPSS, Inc., Chicago, IL, USA).

## Results

**Figure 1:**
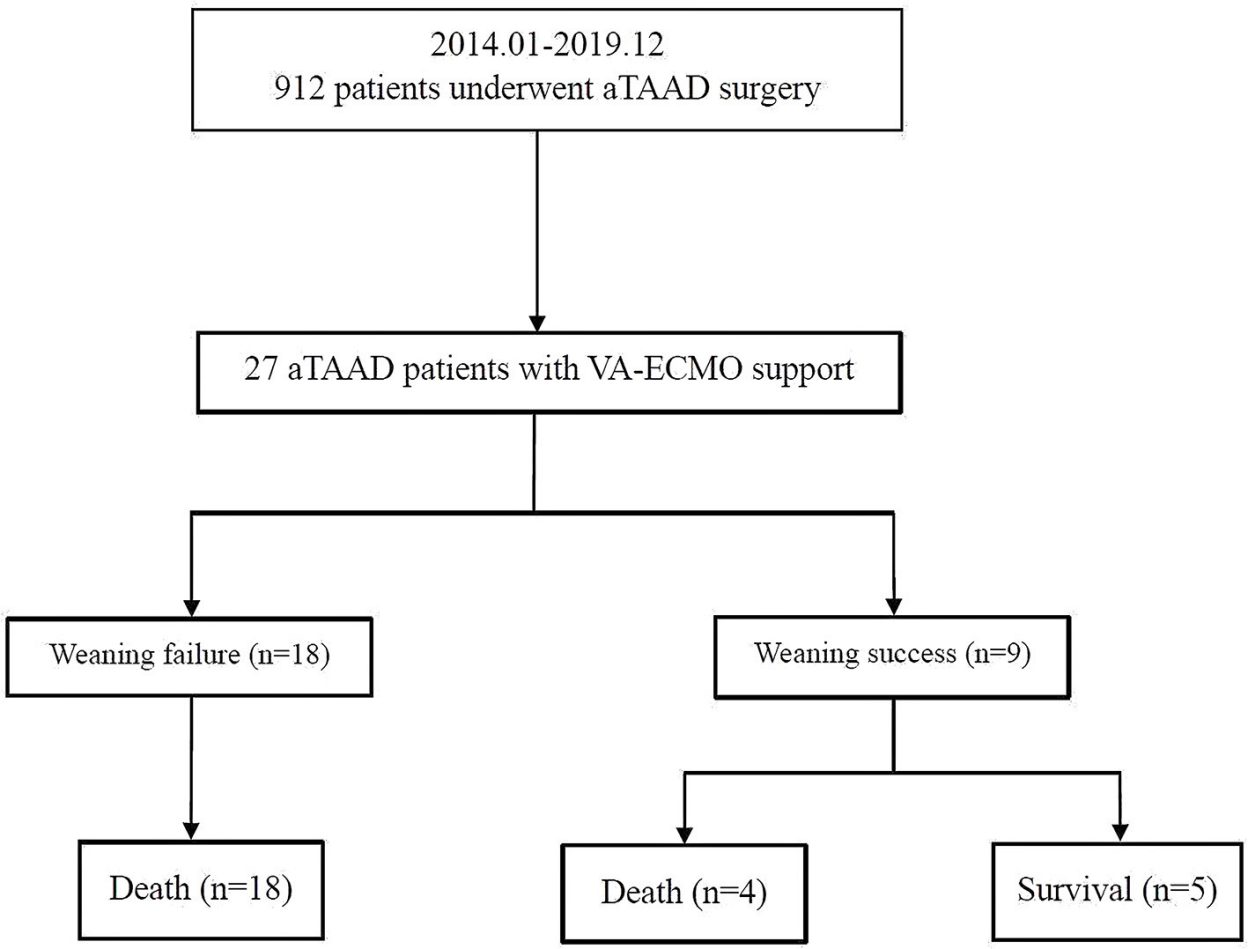
Enrollment, allocation, and follow-up of aTAAD patients who received VA-ECMO. aTAAD, acute type A aortic dissection; VA-ECMO, veno-arterial extracorporeal membrane oxygenation.

Twenty-seven patients, who were supported with VA-ECMO following aTAAD surgery, were enrolled in the study between January 2014 and December 2019 (Figure 1). The mean age of the patients was 53 ± 14 years and 81% were male. Demographics, comorbidities, laboratory tests, and clinical manifestations of the successful group and failed weaning group are shown in Table 1. The preoperative CK-MB level in the failed group was significantly higher than that in successful group (14 [6–30] vs. 55 [28–138] U/L; *p* < 0.01). Age and preoperative cardiac troponin T (cTnT), lactate dehydrogenase (LDH), and creatinine (Cr) levels were higher in the failed group than in the successful group (45 ± 17 vs. 56 ± 12 years; 0.03 [0.01–0.68] vs. 0.97 [0.05–4.57] ng/mL; 253 [175–399] vs. 355 [280–579] U/L; 76 ± 37 vs. 160 ± 73 μmol/L), but the differences between the two groups were not statistically significant (*p* > 0.05).

**Table 1:** Demographic and clinical characteristics of ECMO patients prior to surgery.

**Variables**	**Total (*n* = 27)**	**Wean-from ECMO**		***p*-value**
		**Success (*n* = 9)**	**Failure (*n* = 18)**	
Age, year	53 ± 14	45 ± 17	56 ± 12	0.06
Male, *n* (%)	22 (81)	7 (78)	15 (83)	1.00
BMI (kg/m^2^)	25.59 ± 3.49	25.45 ± 5.06	25.66 ± 2.58	0.91
Comorbidities, *n* (%)				
Hypertension	24 (89)	6 (67)	18 (100)	0.03
Diabetes mellitus	1 (4)	0 (0)	1 (6)	1.00
COPD	5 (19)	1 (11)	4 (22)	0.64
Marfan syndrome	1 (4)	0 (0)	1 (6)	1.00
Atrial fibrillation	1 (4)	0 (0)	1 (6)	1.00
Clinical manifestation, *n* (%)				
Coronary artery involvement	11 (41)	2 (22)	9 (50)	0.20
Cardiac tamponade	2 (7)	0 (0)	2 (11)	0.54
Aortic valve regurgitation	3 (11)	1 (11)	2 (11)	1.00
Previous cardiac surgery, *n* (%)	5 (19)	1 (11)	4 (22)	0.64
Laboratory tests				
CK-MB level (U/L)	38 (15, 61)	14 (6, 30)	55 (28, 138)	0.01
cTnT (ng/ml)	0.60 (0.02, 3.45)	0.03 (0.01, 0.68)	0.97 (0.05, 4.57)	0.09
BNP (pg/ml)	732 (262, 2,559)	487 (196, 2,256)	1,358 (306, 2,879)	0.46
Hb (g/L)	131 ± 25	126 ± 19	134 ± 28	0.50
PLT (×10^9^/L)	195 ± 67	226 ± 65	179 ± 63	0.09
WBC (×10^12^/L)	13 ± 5	12 ± 5	13 ± 5	0.53
Neutrophils (%)	80 ± 11	75 ± 13	83 ± 9	0.09
TBIL (μmol/L)	21 ± 29	17 ± 8	24 ± 35	0.60
DBIL (μmol/L)	10 ± 25	5 ± 1	12 ± 30	0.49
ALB (g/L)	39 ± 4	39 ± 5	39 ± 4	0.83
ALT (U/L)	39 (24, 70)	30 (14, 45)	44 (25, 89)	0.12
AST (U/L)	57 (23, 152)	28 (20, 68)	94 (27, 173)	0.08
LDH (U/L)	302 (250, 520)	253 (175, 399)	355 (280, 579)	0.06
GFR (ml/min/1.73 m^2^)	72 ± 23	76 ± 31	69 ± 19	0.47
Cr (μmol/L)	132 ± 75	76 ± 37	160 ± 73	0.05
BUN (mmol/L)	8 (4, 10)	5 (4, 9)	9 (5, 10)	0.18
Lac (mmol/L)	1.3 (1.0, 1.9)	1.1 (1, 1.7)	1.3 (1.1, 2.0)	0.43
EuroSCORE	7 ± 3	6 ± 3	8 ± 3	0.10
LVEF (%)	60 ± 8	58 ± 8	62 ± 8	0.20
Time from onset to hospital (h)	11 ± 6	9 ± 6	11 ± 6	0.36
Time from onset to operation (h)	22 ± 14	22 ± 16	22 ± 13	0.93

*Continuous data are presented as the mean (SD) or median (IQR). Categorical data are presented as counts (%)*.

*ECMO, extracorporeal membrane oxygenation; BMI, body mass index; COPD, chronic obstructive pulmonary disease; CK-MB, creatine kinase isoenzyme; cTnT, cardiac troponin T; BNP, brain natriuretic peptide; Hb, hemoglobin; PLT, platelet; WBC, white blood cell; TBIL, total bilirubin; DBIL, direct bilirubin; ALB, albumin; ALT, alanine aminotransferase; AST, aspartate aminotransferase; LDH, lactate dehydrogenase; GFR, Glomerular filtration rate; Cr, serum creatinine; BUN, blood urea nitrogen; Lac, lactate; EuroSCORE, European System for Cardiac Operative Risk Evaluation; LVEF, left ventricular ejection fraction*.

Peri-operative details of the 27 patients who were supported with VA-ECMO are summarized in Table 2. Coronary artery bypass grafting was performed in ten patients because of poor cardiac function caused by coronary artery dissection. Although there were no significant difference in operation mode, operation time, CPB time, aortic cross clamp time, or deep hypothermic circulatory arrest time between the two groups, the CPB and aortic cross clamp times in the failed group were longer than those in the successful group (235 ± 83 vs. 290 ± 129 min; 94 ± 19 vs. 120 ± 46 min; *p* > 0.05). Postoperative peak CK-MB, cTnT, and LDH levels were all significantly higher in patients who failed withdrawal of VA-ECMO (134 ± 92 vs. 300 ± 161 U/L; 4.0 [0.9–7.1] vs. 19.5 [6.6–29.9] ng/mL; 1,178 ± 669 vs. 2,468 ± 1,181 U/L, respectively; *p* < 0.01). Postoperative peak lactate levels were also higher in the successful group than in the failed group (19 ± 3 vs. 16 ± 4 mmol/L; *p* < 0.05).

**Table 2:** Intraoperative and postoperative clinical characteristics.

**Variables**	**Total (*n* = 27)**	**Wean-from ECMO**		***p*-value**
		**Success (*n* = 9)**	**Failure (*n* = 18)**	
Intraoperative conditions				1.0
Ascending aorta+arch+ET (*n*)	17	6	11	
Bentall (*n*)	4	1	3	
Bentall+hemiarch (*n*)	2	1	1	
Bentall+arch+ET (*n*)	4	1	3	
Coronary artery bypass graft (*n*)	10	4	6	
Mitral valve surgery (*n*)	1	0	1	
Operation time (h)	8.58 ± 2.25	8.50 ± 1.87	8.63 ± 2.47	0.90
CPB time (min)	271 ± 117	235 ± 83	290 ± 129	0.26
Aortic cross clamp time (min)	111 ± 40	94 ± 19	120 ± 46	0.12
DHCA time (min)	20 ± 9	22 ± 4	18 ± 11	0.29
Post-ECMO support conditions				
Perioperative blood transfusion (U)	17 ± 5	20 ± 6	16 ± 4	0.37
Peak CK-MB (U/L)	244 ± 161	134 ± 92	300 ± 161	0.01
Peak cTnT (ng/ml)	7.7 (4.0, 21.8)	4.0 (0.9, 7.1)	19.5 (6.6, 29.9)	0.01
Peak BNP (pg/ml)	3,400 (2,500, 7,845)	3,400 (2,525, 7 500)	3,429 (2,350, 8,671)	0.71
Peak lactate (mmol/L)	18 ± 3	16 ± 4	19 ± 3	0.02
Peak TBIL (μmol/L)	41 (30, 58)	38 (32, 58)	44 (28, 63)	0.78
Peak DBIL (μmol/L)	24 (19, 44)	23 (14, 41)	35 (19, 45)	0.40
Peak ALT (U/L)	334 (163, 1,205)	220 (179, 1,002)	372 (106, 1,615)	0.82
Peak AST (U/L)	584 (318, 1,720)	340 (273, 1,694)	959 (379, 2,624)	0.28
Peak LDH (U/L)	2,038 ± 1,197	1,178 ± 669	2,468 ± 1,181	0.01
Peak GFR (ml/min/1.73 m^2^)	22 (18, 30)	24 (15, 61)	22 (19, 26)	0.67
Peak BUN (mmol/L)	19 ± 10	21 ± 4	18 ± 8	0.45
Peak Cr (mmol/L)	263 (185, 312)	220 (129, 383)	263 (192, 324)	0.78
Peak PCT (ng/ml)	24 (14, 56)	29 (13, 40)	21 (15, 76)	0.53

*ECMO, extracorporeal membrane oxygenation; ET, elephant trunk; CPB, cardiopulmonary bypass; DHCA, deep hypothermic circulatory arrest; CK-MB, creatine kinase isoenzyme; cTnT, cardiac troponin T; BNP, brain natriuretic peptide; TBIL, total bilirubin; DBIL, direct bilirubin; ALT, alanine aminotransferase; AST, aspartate aminotransferase; LDH, lactate dehydrogenase; GFR, glomerular filtration rate; Cr, serum creatinine; BUN, blood urea nitrogen; PCT, procalcitonin*.

**Figure 2:**
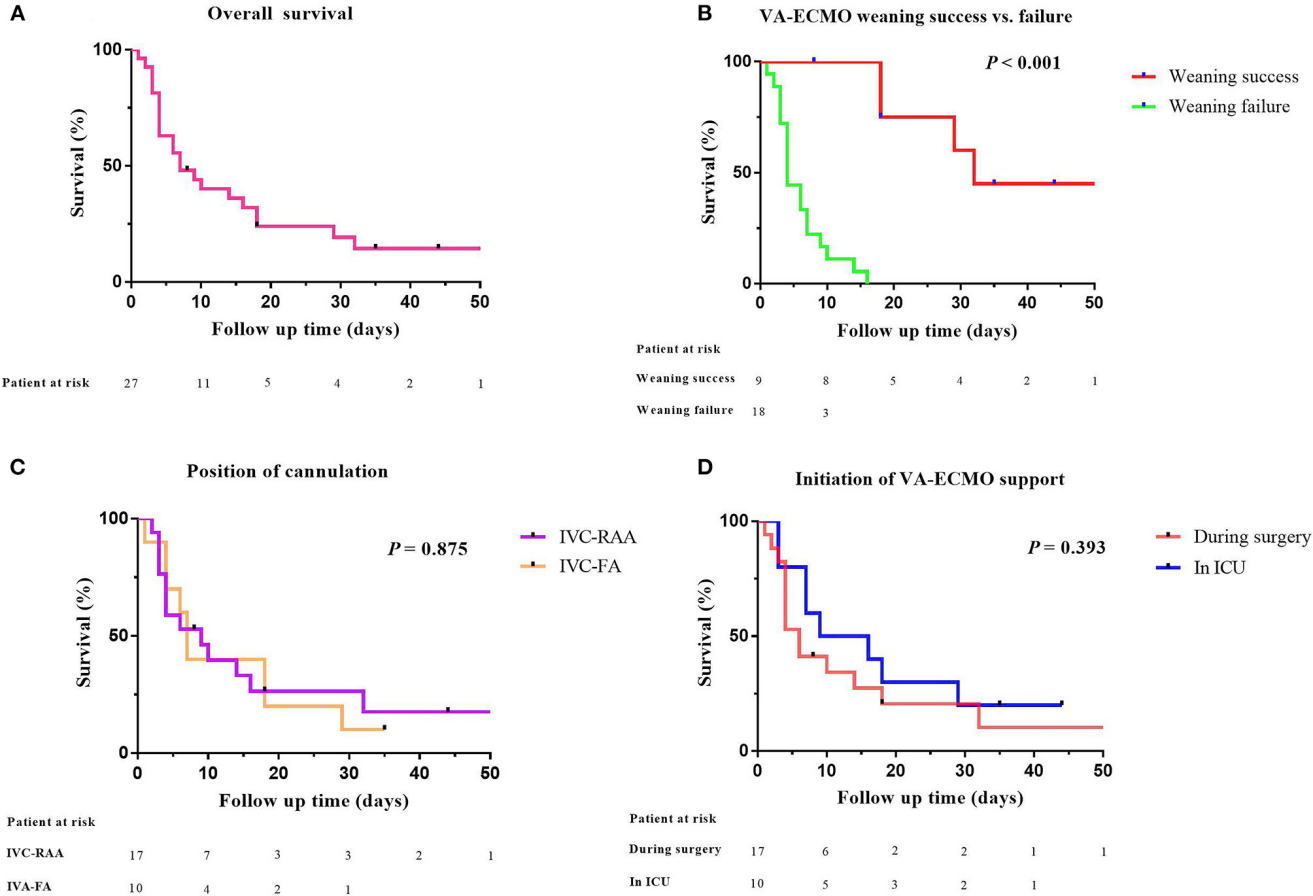
Comparative survival in aTAAD patients after VA-ECMO support. **(A)** Kaplan–Meier analysis of overall survival in aTAAD patients supported by VA-ECMO from 2014 to 2019 (*n* = 27); **(B)** VA-ECMO weaning success or failure; **(C)** position of cannulation; **(D)** Initiation of VA-ECMO support (one patient in the successful weaning group was discharged after 96 days of follow-up. The abscissa was set to 50 days, one data point is outside the axis limits). aTAAD, acute type A aortic dissection; VA-ECMO, veno-arterial extracorporeal membrane oxygenation.

Seventeen patients received VA-ECMO support in the operating room because of difficulty in weaning from CPB, and ten patients had VA-ECMO initiated in the cardiac surgery ICU because of refractory postoperative cardiogenic shock. No progression of aortic dissection was observed during VA-ECMO support. After a median period of 192 h of support, nine patients (33%) were successfully weaned from VA-ECMO support and five patients (19%) survived to hospital discharge. One patient had residual right lower limb movement disorder. The in-hospital mortality rate was 81% (22 patients). The follow-up survival rate of aTAAD patients who required perioperative VA-ECMO support was relatively low (Figure 2).

Additionally, patients who were successfully weaned from VA-ECMO had a longer mechanical ventilation time (16 [8–31] vs. 3 [1–7] days; *p* < 0.01), a longer ICU stay (16 [11–35] vs. 3 [2–5] days; *p* < 0.01) and a longer hospital stay (29 [18–40] vs. 4 [3–8] days; *p* < 0.01), compared with patients who failed weaning from VA-ECMO. No patient was lost to follow-up during this period. Details of VA-ECMO implementation and outcomes are presented in Table 3.

**Table 3:** ECMO implementation and clinical outcomes.

**Variables**	**Total (*n* = 27)**	**Wean-from ECMO**		***p*-value**
		**Success (*n* = 9)**	**Failure (*n* = 18)**	
Initiation of VA-ECMO support (*n*)				0.68
During surgery	17	5	12	
In ICU	10	4	6	
Position of cannulation (*n*)				0.41
IVC-FA	11	5	6	
IVC-RAA	16	4	12	
ECMO duration (h)	82 (46, 192)	192 (111, 327)	55 (23, 95)	0.01
MV time (d)	6 (2, 14)	16 (8, 31)	3 (1, 7)	0.01
CRRT, *n* (%)	19 (70)	6 (67)	13 (72)	0.77
Major complications, *n* (%)				
Bleeding	10 (37)	1 (11)	9 (50)	0.23
Tamponade	0 (0)	0 (0)	0 (0)	1.00
Neurological complications	4 (15)	2 (22)	2 (11)	0.58
VT/VF	3 (11)	0 (0)	3 (17)	0.53
Infection	1 (4)	1 (11)	0 (0)	1.00
MODS	4 (15)	0 (0)	4 (22)	0.27
ICU stay (d)	6 (3, 14)	16 (11, 35)	3 (2, 5)	0.01
Hospital stay (d)	7 (4, 18)	29 (18, 40)	4 (3, 8)	0.01
Mortality, *n* (%)	22 (81)	4 (44)	18 (100)	0.01

*VA-ECMO, veno-arterial extracorporeal membrane oxygenation; ICU, intensive care unit; IVC, inferior vena cava; FA, femoral artery; RAA, right axillary artery; MV, mechanical ventilation; CRRT, continuous renal replacement therapy; VT, ventricular tachycardia; VF, ventricular fibrillation; MODS, multiple organ dysfunction syndromes*.

## Discussion

In this single center study, 27 out of 912 aTAAD patients received VA-ECMO and achieved barely satisfactory results. An intra-aortic balloon pump cannot be used in aTAAD patients and VA-ECMO may be the only viable extracorporeal life support technique for aTAAD patients difficult to wean from CPB or with postoperative cardiogenic shock. Despite its higher in-hospital mortality and postoperative complications, VA-ECMO can be considered as salvage treatment in patients after aTAAD surgery.

aTAAD is not only a morphological abnormality of the aortic wall, but also involves hemodynamic changes that affect cardiac function and the blood supply of important organs, together with a systemic inflammatory reaction caused by dissection. Improvements in surgical, CPB and cerebral protection techniques, together with better perioperative management strategies (nitrogen oxide, continuous renal replacement therapy, and VA-ECMO), have led to a gradual decrease in the mortality rate of aTAAD, with mortality rates reported to be 15–30% (15, 16). VA-ECMO can provide temporary mechanical circulatory support and allow time for etiological treatment (6). Previous studies have shown that the in-hospital mortality of patients who receive VA-ECMO assistance after aTAAD surgery is still high (Table 4) (17–20). Nevertheless, there have been no randomized controlled studies. Additionally, successful weaning does not mean better survival, since 20–65% of patients weaned from VA-ECMO support do not survive to discharge (21). In our series, the successful weaning rate and mortality following VA-ECMO were 33.3 and 81.5%, respectively. The main causes of this difference may be associated with the onset time of aTAAD, the basic condition, surgical strategies, the indication of VA-ECMO, and cannulation strategies.

**Table 4:** Studies concerning the role of VA-ECMO in aTAAD patients.

**Reference**	**Study design**	**Sample size**	**Time of VA-ECMO implantation**	**Approach of cannulation**	**Wean from VA-ECMO**	**Mortality**
Lin et al. (17)	Retrospective study	20	Postoperative	IVC-RAA is preferred	65%	65%
Sultan et al. (18)	Retrospective study	35 (31 open surgery)	27 during surgery	No mention	No mention	89.7%
			8 after surgery			
Wang et al. (19)	Retrospective study	7	6 during surgery	IVC-FA	100%	14.3%
			1 after surgery			
Mariscalco et al. (20)	Retrospective study	62	46 during surgery	19 central arterial cannulation	37%	74%

*VA-ECMO, veno-arterial extracorporeal membrane oxygenation; aTAAD, acute type A aortic dissection; IVC, inferior vena cava; FA, femoral artery; RAA, right axillary artery*.

Several factors may be associated with VA-ECMO weaning failure. Younger age, lower preoperative CK-MB levels, reduced postoperative blood transfusion, higher antegrade cannulation rates, lower lactate levels, lower rates of continuous renal replacement therapy, and organ ischemia have all been shown to influence survival of aTAAD patients after VA-ECMO support (10, 17). We also found that preoperative CK-MB levels were significantly higher in the failed group than in the successful group. Although patients who failed weaning were older and had higher preoperative cTnT levels before CPB than the successfully weaned group, these differences were not statistically significant. Additionally, differences in perioperative blood transfusion, rate of antegrade cannulation, and rate of continuous renal replacement therapy did not reach statistical significance. Postoperative peak levels of cTnT, CK-MB, LDH, and blood lactate were, however, significantly higher in patients who failed weaning from VA-ECMO. CTnT and CK-MB are commonly used as indicators of acute myocardial infarction. Higher cTnT and CK-MB levels after 24–48 h of VA-ECMO support may be associated with poor prognosis. LDH, a key enzyme that regulates the conversion of pyruvate to lactic acid during anaerobic glycolysis, is widely distributed in the cytoplasm, and in mitochondria of the heart, liver, skeletal muscle, and other tissues and cells. When ischemic myocardial injury occurs, the damaged myocardial cell membranes rupture and LDH, which can also be used as a diagnostic marker of myocardial injury (22), is released into the blood serum. Failure to successfully wean from VA-ECMO may be attributable to more severe organ ischemia, including myocardial ischemia, liver and kidney injury, caused by aortic dissection before VA-ECMO was started.

In terms of cannulation strategies, peripheral cannulation is minimally invasive and is the routine pathway for most adult patients. Although femoral artery-femoral vein catheterization is easier and faster, we preferred to cannulate in the right axillary artery because of residual aortic dissection in aTAAD patients. Axillary artery cannulation is, however, technically more difficult than the femoral artery and there is also a risk of hyperperfusion of the ipsilateral arm and brain (23). We compared outcomes of patients who were subjected to the two different cannulation strategies and found there was no significant difference in prognosis between the two groups.

Complications of VA-ECMO support following aTAAD surgery include bleeding, cerebral dysfunction, malperfusion of vital organs, and infection. In our study, death usually occurred soon after surgery and major bleeding was the most common cause of death in patients who could not be successfully weaned from VA-ECMO. Causes of postoperative bleeding may be multifactorial (24–26). Systemic inflammatory reaction caused by dissection, longer CPB time, massive blood transfusion during surgery, hypothermia and postoperative VA-ECMO assistance all result in impaired postoperative coagulation and bleeding. Precise management and proper hemostasis, including bedside thromboelastography, reasonable infusion of fresh frozen plasma, platelets, fibrinogen, and prothrombin complex, are, therefore, very important during surgery.

Our study has several limitations. Firstly, the study was a single-center, retrospective study. Secondly, because of the rarity of aTAAD with VA-ECMO support, the sample size was too small and the follow-up time was relatively short, which meant that detailed analysis of risk factors was not possible. In the future, multicenter studies, with large patient populations, are needed to optimize management strategies and improve outcomes in this rare but complex cardiac emergency.

## Conclusions

This study showed that the use of VA-ECMO in aTAAD patients is a viable salvage strategy, despite the relatively high rate of complications and mortality. VA-ECMO could provide a bridge-to-recovery for aTAAD patients with refractory cardiogenic shock.

## Data Availability Statement

The raw data supporting the conclusions of this article will be made available by the authors, without undue reservation.

## Author Contributions

J-yH, G-wT, and ZL contributed to study design. Y-xS, J-yH, HW, HL, J-lZ, and Jc-L contributed to enrollment of participants. J-lZ and XL contributed to study management and data collection. J-yH and C-sW contributed to manuscript writing. G-wT and ZL contributed to data analyses and manuscript revision. All authors have read and approved the final manuscript.

## Conflict of Interest

The authors declare that the research was conducted in the absence of any commercial or financial relationships that could be construed as a potential conflict of interest.
